# Bioherbicidal potential of plant species with allelopathic effects on the weed *Bidens bipinnata* L.

**DOI:** 10.1038/s41598-022-16203-5

**Published:** 2022-08-05

**Authors:** Robson Willian Nunes Lopes, Estefenson Marques Morais, Julian Junio de Jesus Lacerda, Francisca Diana da Silva Araújo

**Affiliations:** Campus Professora Cinobelina Elvas, Federal University of Piauí, Bom Jesus, PI 64900-000 Brazil

**Keywords:** Plant sciences, Ecology

## Abstract

Plant species with allelopathic effects against weeds have emerged as a potential strategy for the development of ecologically friendly bioherbicides. In this study, the allelopathic effects of the plant species *Dipteryx lacunifera* Ducke, *Ricinus communis* L., *Piper tuberculatum* Jacq., and *Jatropha gossypiifolia* L. on the weed *Bidens bipinnata* L. were investigated. In vitro bioassays revealed that aqueous extracts of selected plant species were able to inhibit seed germination and seedling growth of *B. bipinnata*, highlighting the strongest allelopathic effect evidenced by *R. communis*. The phytotoxicity of the aqueous extracts was evaluated in pot experiments, which indicated that the foliar application of *R. communis* and *P. tuberculatum* extracts on *B. bipinnata* plants caused yellowing of leaves, affecting the chlorophyll content and reducing growth. The discrimination of the plant extracts by attenuated total reflectance Fourier transform mid-infrared (ATR FT-MIR) spectroscopy combined with principal component analysis (PCA) indicated the presence of allelochemical compounds, such as phenolics and terpenoids, which may be associated with allelopathic activity. Overall, this study provides valuable information about the substantial allelopathic inhibitory effects of the plant species *R. communis* and *P. tuberculatum* on the weed *B. bipinnata*, which may be used for the development of eco-friendly bioherbicides.

## Introduction

Weeds are one of the main factors limiting food production in agricultural systems around the world. Factors such as competition for water, light, nutrients and space, triggered by weeds, corroborate the reduction in productivity or quality of the harvested product when they grow simultaneously with the crops^1^. The impacts caused in agricultural areas where there is no adequate weed control have been reported for several crops, such as *Gossypium hirsutum* L.^2^ and *Glycine max* (L) Merr.^3^, among others^4,5^.

*Bidens bipinnata* L. (Asteraceae) is a weed widely distributed in Southeastern Asia, Europe, North and South America, and the Pacific Islands^6^, occurring in all regions of Brazil^7^. It is an annual herb that adapts to both wet and dry situations and generally grows well in moist, fertile environments^6^. In addition to competing with the main crop^8^ and serving as a host for pests and diseases^9^, it can cause productivity losses in agricultural crops, which makes it an aggressive invasive species.

Many weed management methods in different systems have been developed over time^10–12^, among which the use of herbicides and manual and mechanical removal are the most explored control methods. Traditionally, weeds are controlled through the application of synthetic herbicides due to their high efficiency; however, their indiscriminate use promotes negative impacts on crops, human health, and the environment, in addition to the development of resistant weeds^13^. The challenges associated with the use of herbicides make the development of new ecologically friendly methods imperative.

Research on allelopathic plant species is increasingly in evidence from the perspective of their manipulation for practical applications in agriculture in weed control^14–16^. Allelopathy refers to the ability to exert stimulatory or inhibitory effects by one plant, including microorganisms, on another through allelochemicals produced and released into the environment^17^. Allelochemicals related to allelopathy belong to the classes of phenolics, terpenoids, and alkaloids^16,18^. Although research on allelochemicals used as eco-friendly bioherbicides has been ongoing for a long time, there are very few products derived from allelochemicals on the market.

The mode of action of allelochemicals may include germination inhibition, interference with root or seedling growth, reduction in photosynthetic rate and chlorophyll content, interference with enzymatic activity, reduction in mineral absorption and carbon flux, inhibition of cell division, protein synthesis and respiration^19^. In this sense, allelopathy emerges as an ecologically friendly and sustainable alternative for weed control and can be used in different ways, such as through allelopathic plant extracts alone or in combination with reduced synthetic herbicide doses, mix cropping/intercropping, crop rotation, incorporation into the soil, cover crop, or even green manures^20,21^.

Few studies have been carried out to investigate the allelopathy between plant species and *B. bipinnata*. Previous reports described the interaction with *Chrysanthemum boreale* L.^22^ and some crop species^8^. Therefore, in our study, we evaluated the allelopathic effects of the plant species *Dipteryx lacunifera* Ducke, *Ricinus communis* L., *Piper tuberculatum* Jacq., and *Jatropha gossypiifolia* L. on the weed *B. bipinnata* to investigate their bioherbicidal potential. For this purpose, in vitro seed germination bioassays and pot experiments were performed using aqueous plant extracts to simulate natural conditions. Attenuated total reflectance Fourier transform mid-infrared (ATR FT-MIR) spectroscopy, combined with principal component analysis (PCA), was used to discriminate the extracts.

## Results and discussion

### Effects of aqueous plant extracts on germination and early growth of *B. bipinnata* by in vitro bioassays

Seed germination and seedling growth of *B. bipinnata* were investigated after treatment with DT, RC, PT, and JG aqueous extracts to explore the allelopathic effects of these plant species. The pH of the aqueous extracts corresponded to 6.62 for DL, 5.59 for RC, 7.20 for PT, and 7.42 for JG, with no significant difference in pH values between DL and RC extracts or between PT and JG extracts; however, the pH of DL and RC extracts differed significantly (p < 0.05) from PT and JG. Germination and early development of seedlings are affected by extreme pH values when very acidic (below 4) or very alkaline (above 10). In this context, the pH values of the extracts did not influence the germination of *B. bipinnata* seeds, considering that pH values between 6.0 and 7.5 are considered ideal for the biochemical and nutritional processes of plants^23^.

**Figure 1:**
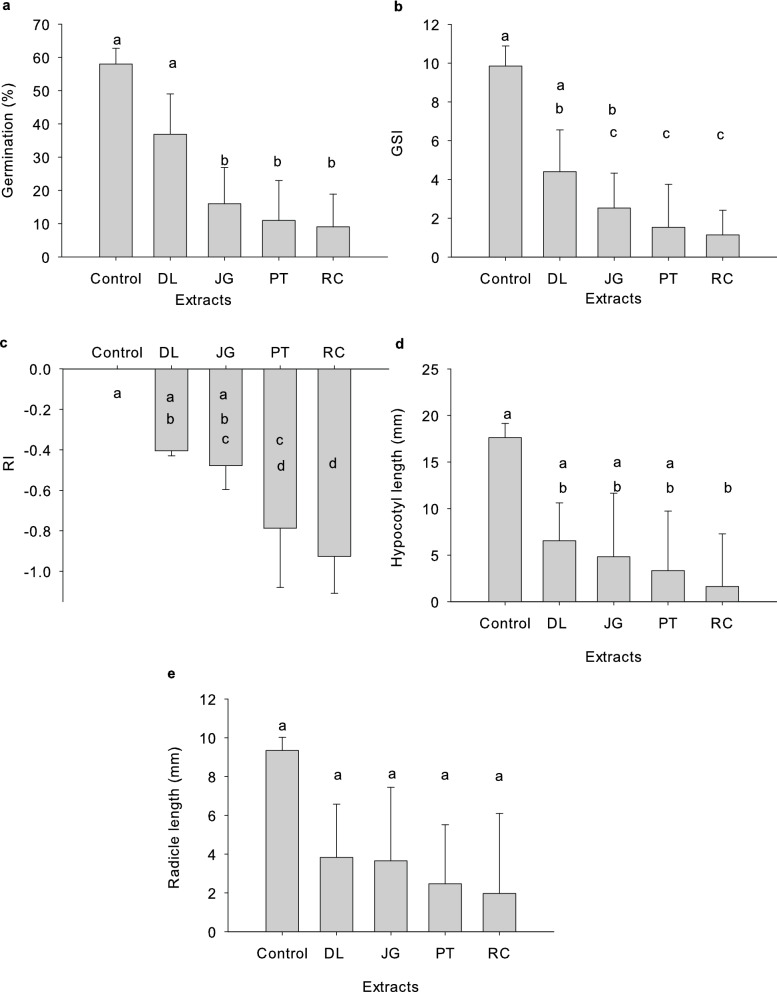
Germination (**a**), germination speed index (GSI) (**b**), allelopathic effect response index (RI) (**c**), and hypocotyl (**d**) and radicle length (**e**) of *B. bipinnata* treated with *D. lacunifera* (DL), *R. communis* (RC), *P. tuberculatum* (PT), and *J. gossypiifolia* (JG) extracts. The bars in the figure represent the standard deviation of the repetitions. Treatments with the same letter are statistically similar by Dunn's test at the 0.05 level.

The germination percentage and germination speed index (GSI) of *B. bipinnata* seeds were significantly affected by botanical extracts (p < 0.001) (Tables S1, S2). The treatments of *B. bipinnata* seeds with RC, PT, and JG extracts were statistically similar to each other and different from the control treatment for the variable germination percentage, while the treatments with DL extracts did not differ significantly from the control (Table S3 and Fig. 1a). The decrease in germination occurred with increasing concentrations of the extracts, indicating an inversely proportional relationship between concentration and germination, with significant differences between concentrations (Tables S4 and S5).

**Figure 2:**
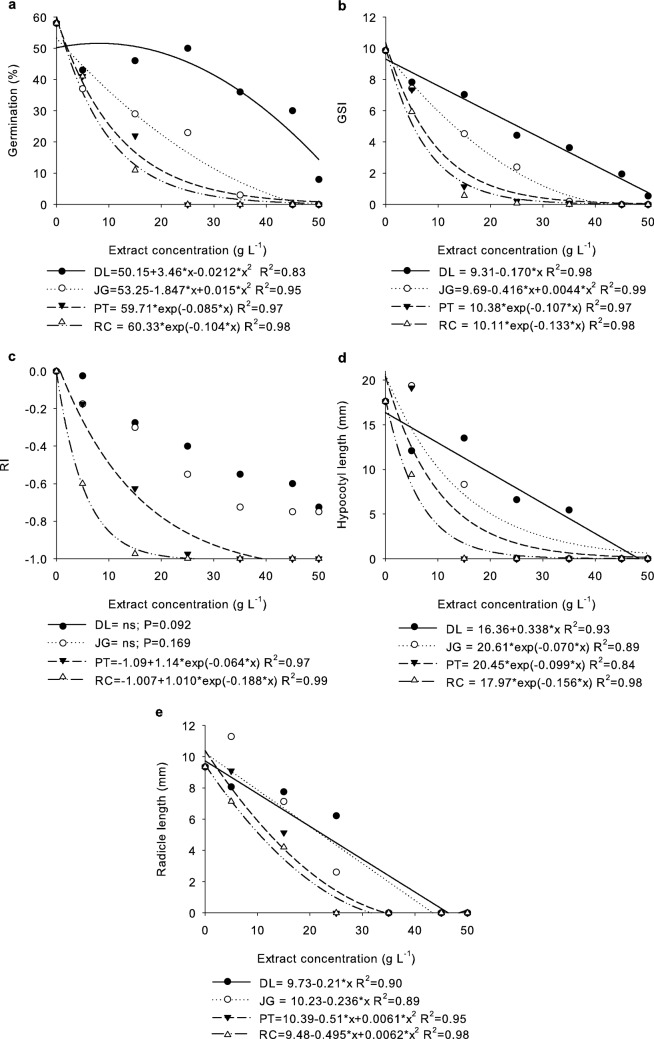
Germination (**a**), germination speed index (GSI) (**b**), allelopathic effect response index (RI) (**c**), and hypocotyl (**d**) and radicle length (**e**) of *B. bipinnata* submitted to treatment with different concentrations of *D. lacunifera* (DL), *R. communis* (RC), *P. tuberculatum* (PT), and *J. gossypiifolia* (JG) extracts. ns: there is no significant difference.

The dose–response curves showed exponential decay for the RC and PT extracts and quadratic decay for the JG and DL extracts (Fig. 2a), reaching total inhibition at 25 g L^−1^ for the RC extract and at 45 g L^−1^ for the PT and JG extracts. Although no significant difference was observed between the DL extract and the control (Table S3 and Fig. 1a), this comparison was based on the mean of the measurements, which masks the individual effect of higher extract concentrations that showed a significant differences (p = 0.033, Table S5).

Regarding the GSI, significant differences were observed between the treatments with DL extracts in relation to the RC and PT extracts, which were similar to each other (Table S3 and Fig. 1b). Although there was no significant difference between JG and DL extracts, JG differed from the control treatment. The DL extract, in turn, was similar to the control treatment in the mean comparison; however, despite not promoting complete inhibition of GSI at any of the concentrations evaluated, there was a significant difference between the concentrations of this extract (p = 0.005) (Table S5), with a dose–response curve showing linear decay (Fig. 2b).

The treatments with RC and PT extracts showed dose–response curves for GSI with exponential decay and quadratic decay for JG (Fig. 2b). The RC and PT extracts reached GSI equal to zero at a concentration of 25 g L^−1^ and the JG extracts at 45 g L^−1^. The GSI is proportional to seed vigor, indicating that higher values represent more vigorous seeds^24^. In our study, seed vigor was significantly reduced due to the interference of the phytotoxic allelochemicals contained in the extracts. In addition, the phytotoxic effect was potentiated with the increase in the concentration of extracts, since there was an increase in the concentration of allelochemicals in the solution^25^.

The allelopathic effect response index (RI) demonstrated the inhibitory effects promoted by each of the extracts on the germination of *B. bipinnata* seeds (Table S1), and an RI equal to − 1.0 indicates the maximum efficiency of allelopathic capacity of the extracts^26^. The RIs of treatments with RC and PT extracts were significantly similar to each other and differed from the control treatment (Table S3 and Fig. 1c), presenting dose–response curves with exponential decay and maximum efficiency of allelopathic activity from 25 g L^−1^ (Fig. 2c). On the other hand, the treatment with JG extract was statistically similar to the DL extract, and both did not differ from the control treatment for the two factors: types of extracts (Table S3) and concentrations (Table S5).

The performance of aqueous extracts of RC leaves has been previously demonstrated in inhibiting germination and early development of other plants, such as *Brassica napus* L*.*^27^, *Raphanus sativus* L*.*^27^, and *Triticum aestivum* L.^28^. Hydroalcoholic extracts of RC also completely inhibited the germination of *Lepidum sativum* L. and *Echinochloa crus-pavonis* L. and the seedling growth of *L. sativum*, *Lactuca sativa* L., *Lolium multiflorum* Lam., and *E. crus-pavonis*^29^. Aqueous extracts obtained from PT leaves inhibited the germination of *L. sativa*^30^, and organic extracts from PT fruits inhibited the germination of *Mimosa pudica* L. and *Senna obtusifolia* (L.) H. Irwin & Barneby^31^. A recently published work showed that aqueous extracts of JG leaves affect the germination and early growth of *Cicer arietinum* L. under laboratory conditions^32^. To the best of our knowledge, this is the first report demonstrating the assessment of DL phytotoxic activity.

Differences in seed germination patterns result from effects on membrane permeability, DNA transcription and translation, functioning of secondary messengers, respiration by oxygen sequestration (phenols), the combination of enzymes and receptors or the combination of these factors^33^. The application of aqueous extracts of dry leaves of RC caused a significant reduction in the mitotic index of *L. sativa* and *Cucumis sativus* L. seeds, indicating allelopathic effects on cell division, which resulted in reductions in the germination percentage^34^. In addition, allelochemical compounds can act by inhibiting or delaying the germination process, which is extremely beneficial for weed management since the delay in germination reduces competition between species during the early development of the crop^35^.

The early development of *B. bipinnata* seedlings was measured by hypocotyl and radicle length (Table S1). Regarding hypocotyl length, it was observed that treatments with DL, PT, and JG extracts were statistically similar to the control treatment (Table S3 and Fig. 1d). On the other hand, there was a significant difference between the RC treatment in relation to the control (Fig. 1d), with a significant difference between concentrations (p = 0.002) (Table S5). The dose–response curve for the treatment with RC extracts showed exponential decay (Fig. 2d), with total inhibition from 15 g L^−1^. Although the other extracts did not differ, there was a significant difference in the concentration factor for each extract (Table S5), with PT and JG extracts promoting total inhibition from 15 and 25 g L^−1^, respectively, and dose–response curves with linear decay for DL and exponential for PT and JG (Fig. 2d).

No significant differences were observed in the radicle length of *B. bipinnata* seedlings submitted to treatments with different botanical extracts (Tables S2 and S3, Fig. 1e) in the comparison of means for the extract type factor; however, there were significant differences between extract concentrations (Tables S4 and S5). The dose–response curves showed linear decay for DL and JG and quadratic decay for RC and PT extracts, with total inhibition of radicles from 35 g L^−1^ for DL and JG extracts and from 25 g L^−1^ for RC and PT (Fig. 2e).

During seedling development, root length is more sensitive to the action of allelochemicals; such substances promote the abnormal formation of seedlings with the presence of necrotic tissues typical of the symptoms observed in allelopathy assays^34^. A higher sensitivity of *L. sativa* radicles to allelochemicals from hydroalcoholic extracts of RC was also reported^29^. Organic extracts from PT fruits promoted a significant reduction in the growth of the radicles and shoots of *S. obtusifolia* and *M. pudica*^31^. The concentrations and types of allelochemicals vary according to the part of the plant in which they are produced, and leaves tend to have the highest concentrations, which has already been proven in other studies^25,36^.

### Phytotoxic effects of aqueous plant extracts on *B. bipinnata* by in vivo bioassays in a greenhouse

The phytotoxic effects of DL, RC, PT, and JG were evaluated in pot experiments via foliar application of aqueous extracts using different concentrations. The DL extract caused slight symptoms (grade 3) at concentrations of 37.5 and 150 g L^−1^, characterized by small leaf discolourations in many plants (Table S6), while concentrations of 75 and 300 g L^−1^ were responsible for small changes, with yellow leaves with very slight symptoms (grade 2) on some plants.

In *B. bipinnata* plants treated with RC and PT extracts, it was found that a concentration of 37.5 g L^−1^ provided strong discolouration (grade 5), characterized by chlorosis in many plants and necrotic spots on young leaves. The other concentrations of these extracts (75, 150, and 300 g L^−1^) showed symptoms of medium phytotoxicity (grade 4), with few yellow and wrinkled leaves according to the grade scale^37^. The treatment of *B. bipinnata* plants performed with JG extract did not cause symptoms of phytotoxicity (grade 1), presenting similar results to the control treatment using only water.

To evaluate the phytotoxicity of the extracts on the growth and physiology of *B. bipinnata* seedlings, the height of the plants, as well as the levels of chlorophyll a, b and total, were measured (Table S7). Regarding seedling height, there were significant differences between treatments with different types of extracts (p < 0.001), which also differed from the control treatment (p < 0.001) (Table S8). The treatments with PT extracts differed statistically from the DL and JG extracts and were similar to the RC extract (Table S8), while there was no significant difference between treatments with DL extracts in relation to JG and RC extracts. However, RC and JG were significantly different from each other.

**Figure 3:**
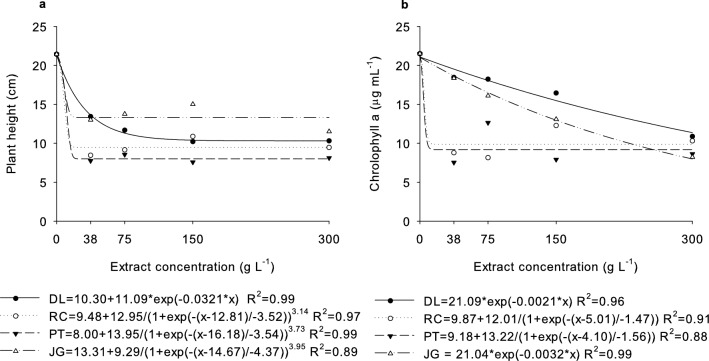
Height (**a**) and chlorophyll a (**b**) of *B. bipinnata* seedlings submitted to increasing doses of *D. lacunifera* (DL), *R. communis* (RC), *P. tuberculatum* (PT), and *J. gossypiifolia* (JG) extracts.

There was no significant difference in height as a function of extract concentrations (p = 0.7869); however, there was a difference between them in relation to 0 g L^−1^ (p < 0.001). The dose–response curves showed exponential decay for DL and sigmoidal decay for RC, PT, and JG extracts (Fig. 3a), reaching the lowest height of 8.45 and 7.75 cm at 37.5 g L^−1^ for RC and PT extracts, respectively, 10.20 cm at 150 g L^−1^ for DL extract, and 11.50 cm at 300 g L^−1^ for JG extract, while the height at 0 g L^−1^ was 21.43 cm.

For the chlorophyll a content of *B. bipinnata* seedlings, there were also significant differences between treatments with the different extracts (p = 0.0177), which differed from the control treatment (p = 0.0109) (Table S8). The DL extract treatment was significantly different from the PT extract, but both were similar to the RC and JG extracts, and the dose–response curves of all treatments showed exponential decay (Fig. 3b). The PT extract reached the lowest chlorophyll a content of approximately 7.54 μg mL^−1^ at 37.5 g L^−1^. Regarding the chlorophyll b content, there were no significant differences between the treatments with the different extracts in relation to the control treatment (Table S9). For total chlorophyll, significant differences were observed only between treatments with RC extracts in relation to the control treatment (Table S9), with no statistical significance for concentrations (Table S10).

Symptoms caused by aqueous extracts of RC and PT in *B. bipinnata* plants may be associated with the presence of allelochemicals in these extracts. Allelopathy can impair plant growth and development through a variety of pathways, such as hormone levels, photosynthesis, respiration, nucleic acid metabolism, and protein synthesis^38^. There are few studies evaluating the phytotoxicity of extracts applied directly to weed leaves in a greenhouse. However, the allelopathic potential of *Leucaena leucocephala* (Lam) de Wit. extracts on the weeds *B. pilosa*, *Desmodium purpureum* (Mill.) Fawc. & Rendle, and *Amaranthus hybridus* L. has already been reported, where concentrations of 100 and 200 g L^−1^ caused reductions in plant growth and leaf blade deformation, but no symptoms of chlorosis were observed in the treated plants^39^.

The application of ethanolic extracts from *Piper aduncum* L. leaves on monocotyledonous, dicotyledonous, and Cyperaceae weed seedlings in greenhouse experiments reduced the photosynthetically active leaf area and showed symptoms of moderate phytotoxicity^40^. Pot experiments using soil mixed with a certain proportion of *Artemisia argyi* H.Lév. & Vaniot powder on seeds of the weeds *Brassica pekinensis* (Lour.) Rupr., *L. sativa*, *Oryza sativa* L., *Portulaca oleracea* L., *Oxalis corniculata* L., and *Setaria viridis* (L.) P.Beauv. have been reported, resulting in reductions in plant height of all species evaluated, as well as a gradual increase in leaf chlorosis^15^.

Based on our results, it was found that RC and PT extracts interfere not only with seed germination but also with *B. bipinnata* growth. We suggest that one of the main mechanisms affected was photosynthesis, considering that the reduction in leaf chlorophyll impaired the photosynthetic rate, resulting in lower plant growth. Reductions in the growth of various weeds and symptoms of leaf chlorosis are attributed to inhibition of chlorophyll synthesis and pathways related to photosynthesis, nitrogen metabolism, and porphyrin metabolism^15,38^.

### Total phenolic content of allelopathic plant extracts

**Figure 4:**
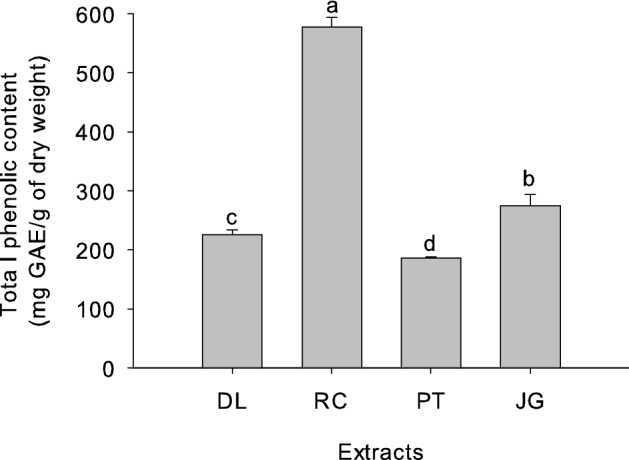
Total phenolic contents of *D. lacunifera* (DL), *R. communis* (RC), *P. tuberculatum* (PT), and *J. gossypiifolia* (JG) extracts. GAE = gallic acid equivalent. The bars in the figure represent the standard deviation of the repetitions (n = 4). Treatments with the same letter are statistically similar by Tukey's test at the 0.05 level.

The mechanism of allelopathy associated with phenolic compounds has been studied extensively^16,18^. To investigate whether these compounds would be involved in the allelopathic activity of DL, RC, PT, and JG extracts, the quantification of total phenols of the aqueous extracts was performed by the Folin-Ciocalteu method, expressed as mg of gallic acid equivalents (GAE)/g of dry weight. The extracts showed significant differences (p=0.0000) in the levels of total phenolics (Fig. 4), with the highest levels observed in the RC extract (577.53 ± 15.87), followed by JG (274.84 ± 18.81) and DL (225.35 ± 8.76), while the lowest content was obtained for the PT extract (186.00 ± 2.56 mg of GAE/g of dry weight).

The highest total phenolic content in the RC extract may be related to the allelopathic activity, since this species was responsible for the greatest inhibitory effects on the germination and growth of *B. bipinnata* (Figs. 1, 2, 3). Phenolic compounds of RC leaf extracts have also been suggested to be responsible for the reduction in the growth and germination rates of *Vigna radiata* L., *Solanum lycopersicum* L., and *Zea mays* L.^41^. They can increase the permeability of the cell membrane, causing the spread of cell contents and increasing lipid peroxidation, causing the slow growth or death of plant tissue and inhibiting nutrient absorption from the environment, affecting the normal growth of plants^42^. Phenolic acids, specifically, can act by inducing an increase in the activity of oxidative enzymes, causing changes in membrane permeability and lignin formation and contributing to the reduction of plant root growth^24^.

The PT extract, although it did not present the highest concentration of total phenols, showed significant allelopathic capacity against *B. bipinnata* in the bioassays (Figs. 1, 2, 3), suggesting that phenolic compounds may act synergistically with other secondary metabolites. Indeed, in addition to phenolic allelochemicals, terpenes and alkaloids may also be involved in allelopathic activity^16,18^, and these classes of compounds have been previously reported in PT leaves^43,44^. Phenolic compounds, terpenes, and alkaloids have also been reported in extracts from different parts of JG, which showed intermediate values of total phenolic content in our study^45,46^. In *D. lacunifera*, flavonoids and terpenes were previously isolated from fruit kernels and shells, respectively^47^, and there are no records of studies on the chemical composition of leaves.

### Discrimination of allelopathic plant extracts by infrared spectroscopy combined with principal component analysis

**Figure 5:**
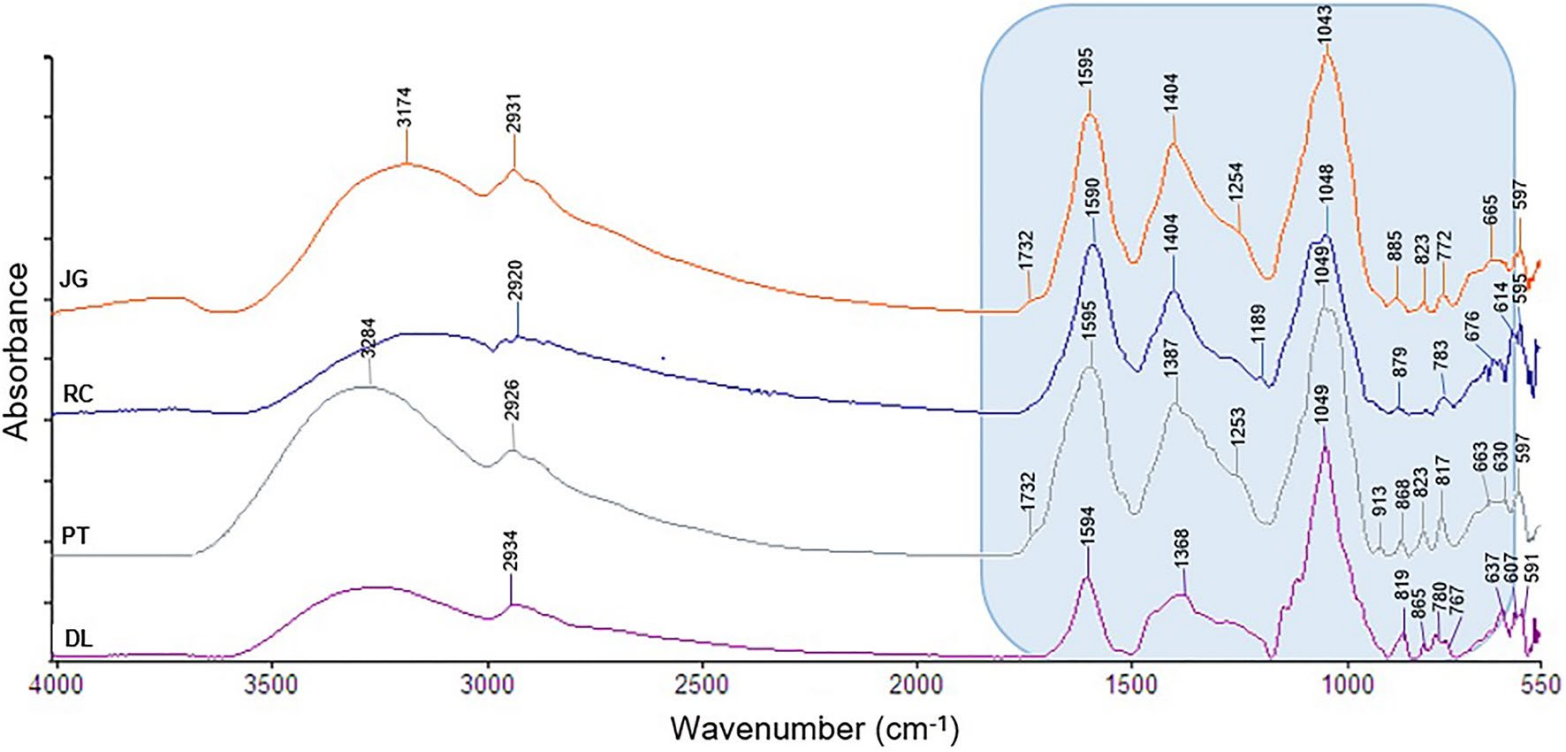
ATR FT-MIR spectra of *D. lacunifera* (DL), *R. communis* (RC), *P. tuberculatum* (PT), and *J. gossypiifolia* (JG) extracts. The fingerprint region of 1800–600 cm^−1^ is colored in blue.

Attenuated total reflectance Fourier transform mid-infrared (ATR FT-MIR) spectroscopy-based metabolomic combined with principal component analysis (PCA) was performed to discriminate the aqueous extracts of DL, RC, PT, and JG and investigate the classes of compounds present on them. Figure 5 shows the infrared spectra of each plant extract, and the main functional group assignments are summarized in Table S8. In general, the ATR FT-MIR spectra of the extracts showed similar absorbance patterns but varied in intensity. The broad band peak in the region of 3500–3100 cm^−1^ is due to the stretching vibration of the O–H bond of alcohol, carboxylic acids, and phenols^48^. Absorption peaks at 3000–2800 cm^−1^ were assigned to asymmetrical and symmetrical stretching of C–H: ν_as_ (CH_3_), ν_s_ (CH_3_), ν_as_ (CH_2_), and ν_s_ (CH_2_) from methyl and methylene groups.

The fingerprint region of 1800–600 cm^−1^ contains many peaks originating from various stretching and deformation modes, making it difficult to identify individual peaks due to their complexity. The peak at 1732 cm^−1^ was attributed to C=O stretching of esters, ketones, and aldehydes. The broad absorption peak at 1650–1500 cm^−1^ was attributed to C=C stretching of alkenes or N–H of amines or amide-containing compounds. The peaks at 1404/1387/1368 cm^−1^ were attributed to the bending vibration of C–H bonds, and the absorption peaks at 1255–1000 cm^−1^ were attributed to C-O stretching. The bands located in the region > 1000 cm^−1^ were attributed to the C − H out-of-plane bending vibration of aliphatic alkenes and aromatic benzene rings^49,50^.

**Figure 6:**
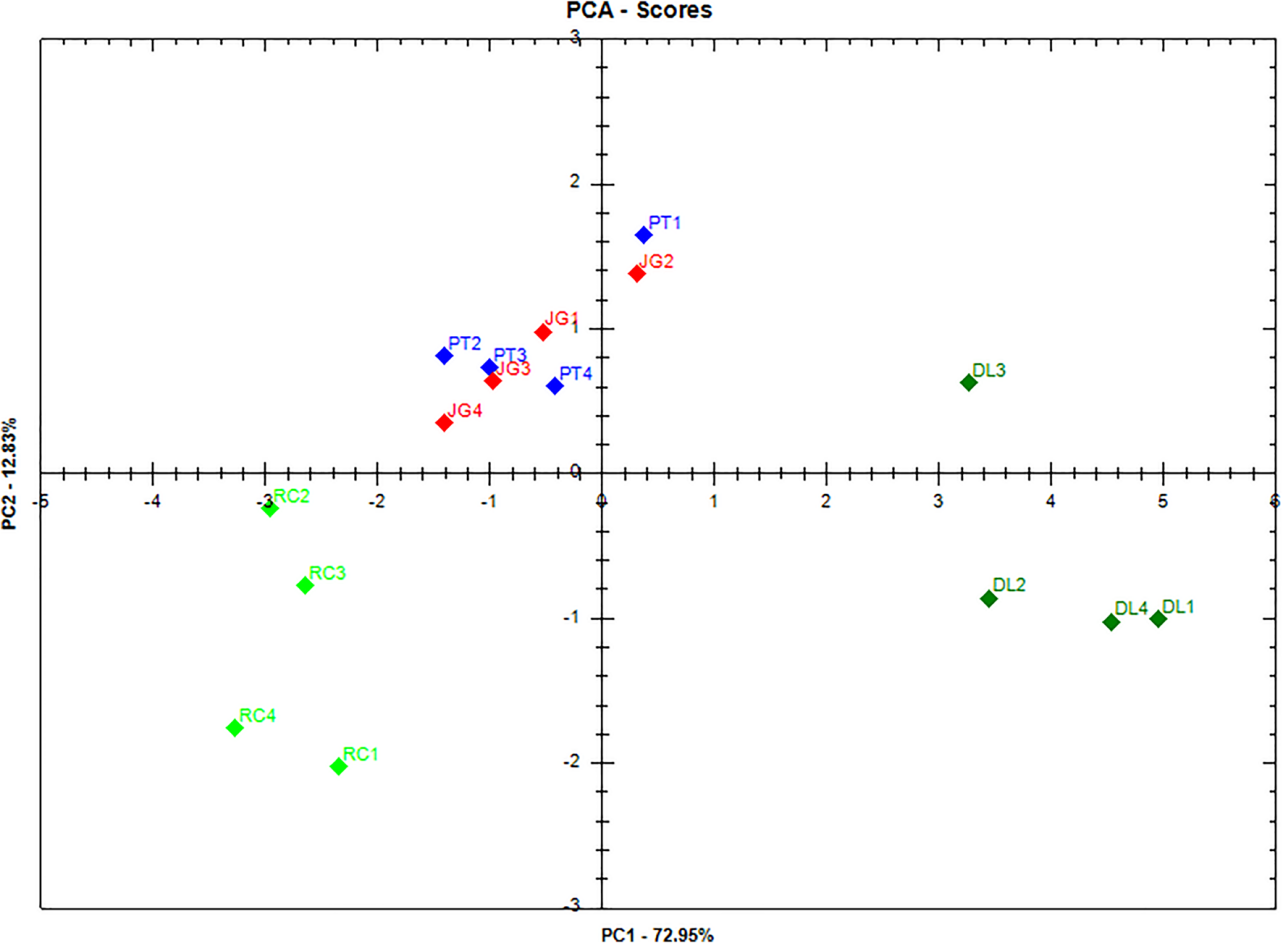
PCA score plot (PC1 × PC2) of *D. lacunifera* (DL), *R. communis* (RC), *P. tuberculatum* (PT), and *J. gossypiifolia* (JG) extracts.

The range between 1800 and 600 cm^−1^ of the infrared spectra was selected for the PCA, as it is the most representative region of the differences present in the spectra. In the PC1 versus PC2 score plot (Fig. 6), representing 85.78% of the total variance, it is possible to observe the separation of the samples into three distinct groups. The samples of DL and RC extracts formed two distinct groups, since they showed a significant separation in the PC1 axis, with positive and negative scores for PC1, respectively. The samples of JG and PT extracts formed a single group, remaining superimposed and located close to the zero value of PC1, indicating intermediate spectral characteristics in relation to the DL and RC extracts. These results may be correlated with the allelopathic activity of these extracts, since the RC extract showed better performance, followed by the JG and PT extracts, with intermediate performance, and the DL extract showed lower activity compared to the others.

The PC1 loading plot (Fig. S1) has as main contributors the negative bands associated with signals at approximately 1732, 1595, 1404, 1200–1025, 1049, and 780–600 cm^−1^, which significantly contributed to the separation of RC extract samples that presented greater intensity than in DL extract samples. On the other hand, the positive bands in PC1 in the region of 780–970 cm^−1^ were more intense in DL extracts. When evaluating the negative region of the PC1 loading plot, it is possible to observe that the functional groups responsible for the discrimination are probably those present in flavonoids and phenolic acids, corroborating the data in the literature that demonstrate the identification of these compound classes in RC leaves, such as gallic acid, quercetin, gentisic acid, rutin, epicatechin, ellagic acid, etc.^51–53^.

The presence of flavonoids can be observed due to the stretching of C=O at approximately 1732 cm^−1^, C=C of aromatics at 1600 cm^−1^, C–O at 1200–1000 cm^−1^, and O–H at 3284–3174 cm^−1^. Phenolic acids can be verified due to stretching of the O–H of carboxylic acid, C=O and aromatic ring, as well as the C − H out-of-plane bending vibration of aromatic benzene ring at < 1000 cm^−1^. Additionally, the presence of terpenes, already reported in RC^52^, can be observed by the stretching of C=C at 1600 cm^−1^ and C–O at 1200–1000 cm^−1^. The higher concentration of these compounds in the RC extract in relation to the other extracts explains the higher allelopathic activity demonstrated by this species. In the positive region of the PC1 loading plot, the peaks of the C−H out-of-plane bending vibration of the aromatic benzene ring contributed to the discrimination of DL samples in relation to RC; however, they appeared at low intensity.

In the PC2 loading plot (Fig. S1), the negative bands at 1775, 1595, 1425–1500, and 800–600 cm^−1^ contributed to the separation of RC extracts, while the positive bands at approximately 1725, 1650, 1400–1100, 1049, and 950–800 cm^−1^ were associated with the separation of sample clustering from the PT and JG extracts. The bands in the negative region of C=C stretching at 1595 cm^−1^ and C−H out-of-plane bending vibration of aromatic benzene ring at 800–600 cm^−1^ may be associated with the higher concentration of phenolic compounds in the RC extract, justifying its greater allelopathic effect compared to PT and JG extracts.

The bands in the positive region of C=C stretching of alkene at 1650 cm^−1^, deformation vibrations of C–H bonds at 1400–1200 cm^−1^, C–O stretching at 1200–1000 cm^−1^, and out-of-plane deformation of C–H bond of alkene at 950–800 cm^−1^ may be associated with a higher concentration of terpenoids in the PT and JG extracts compared to the RC extract. The presence of terpenes in the phytochemical studies of PT^43^ and JG^45,46^ leaves has been reported.

In summary, the plant species DT, RC, PT, and JG demonstrated a potential inhibitory effect on the germination and early growth of *B. bipinnata* via in vitro seed germination bioassays and pot experiments, highlighting the strongest allelopathic effects of the RC and PT species. Metabolomic analysis by infrared spectroscopy combined with PCA indicated the presence of phenolic and terpenoid compounds, which may be associated with the allelopathic activity of the extracts.

Considering the environmental impacts caused by the use of the chemical control method and the growing number of species resistant to the different mechanisms of action of herbicides, the results of our research are promising for the development of ecologically friendly bioherbicides. However, it is important to emphasize the need for further studies to verify the efficiency of the extracts in the emergence of seeds under field conditions and, later, to evaluate the possibility of using these species as raw material for the development of formulations to be inserted in the management of weeds.

## Materials and methods

### Botanical material

Leaves of aerial plant parts in the reproductive phase of DL (latitude 9° 04′ 56.8″ South, longitude 44° 19′ 41.8″ West), RC (latitude 9° 04′ 16.9″ South, longitude 44° 20′ 43.6″ West), PT (latitude 9° 04′ 16.9″ South, longitude 44° 20′ 43.6″ West), and JG (latitude 9° 04′ 26.6″ South, longitude 44° 20′ 31.1″ West), as well as seeds of the weed *B. bipinnata* (latitude 9° 04′ 56.8″ South, longitude 44° 19′ 41.8″ West), were collected in the morning in the region of Bom Jesus-PI, in agricultural cultivation for DL and in native forest for the others. The municipality has a hot and humid climate, classified by Köppen as Aw (tropical climate with summer rains).

The plants were identified by Prof. Gardene Maria de Sousa, and voucher specimens were deposited at the Herbarium Graziela Barroso (Federal University of Piauí, Teresina, PI, Brazil), cataloged under the registration numbers TEPB 32,521 (*B. bipinnata*), TEPB 32,522 (DL), TEPB 32,523 (PT); TEPB 32,524 (RC), and TEPB 32,525 (JG). The plants were registered in the National System for the Management of Genetic Heritage and Associated Traditional Knowledge (SisGen) by n° A0DB8D9 and AE123A5, as recommended by the Brazilian Biodiversity Law (n° 13,123/15).

### Preparation of plant extracts

Freshly harvested leaves of aerial parts of the DL, RC, PT, and JG plants were dried in an oven with forced air circulation at 40 °C until a stable dry mass was obtained. After drying, the plant material was ground in a knife mill to obtain a fine-grained powder-like texture. Thus, 10 g was weighed and transferred to an amber flask, and 200 mL of distilled water was added to each of the extracts, which were homogenized by gently shaking the flask and left to rest at 18 °C for 48 h, obtaining a solution of 50 g L^−1^ stock. The pH of the extracts was measured with a pH meter, and the data were subjected to analysis of variance (ANOVA) and compared by Tukey's test at 5% probability. After this period, the material was filtered and used in in vitro bioassays. For in vivo bioassays, the stock solution was prepared at a concentration of 300 g L^−1^.

### In vitro seed germination bioassays

The plant extracts were diluted to obtain six different concentrations (50, 45, 35, 25, 15, and 5 g L^−1^) using distilled water as a control treatment. For the bioassay, 25 seeds of *B. bipinnata* were distributed in Petri dishes (diameter = 90 mm), previously autoclaved, containing two disks of filter paper. The seeds were previously sterilized by shaking for 5 min in 5% NaOCl, followed by washing with distilled water. Subsequently, the plates were moistened with 7 mL of each extract or distilled water for the control treatment and placed in a BOD incubator at 25 °C and a photoperiod of 12 h for 7 days. The number of germinated seeds was counted daily for 7 days, and germinated seeds that presented primary roots with lengths ≥ 2.00 mm were considered. Primary root and hypocotyl length were determined by measuring from stem base to root tip and stem to plumular hook on 10 seedlings per replicate in each treatment using a digital calliper.

The variables evaluated were germination percentage (PG), radicle length, hypocotyl length, germination speed index (GSI), and allelopathic effect response index (RI), according to their respective equations. PG = (N/A) × 100, where N = number of germinated seeds and A = total number of seeds. GSI = (G1/N1 + G2/N2… Gn/Nn), where G1, G2, and Gn corresponded to the number of seeds germinated in the first, second, and last counts, respectively, and N1, N2, and Nn were the number of days elapsed until the last count^54^. The allelopathic effect response index (RI) was calculated using the equation suggested by Williamson and Richardson (1988)^26^, when T ≥ C, RI = 1 − C/T; when T < C, RI = T/C − 1 (T < C), where C is the control germination speed and T is the treatment germination speed; RI > 0 represents a stimulatory effect, RI < 0 represents an inhibitory effect, and the absolute value is consistent with the allelopathy intensity.

The in vitro bioassay was carried out in a completely randomized design (CRD) with 4 replications, which consisted of a 4 × 6 + 1 factorial, the first factor being the extract type and the second factor the concentration. The Shapiro–Wilk test was performed to test the normality of the data. Due to the lack of adjustment of the residuals to the normal distribution, the Kruskal–Wallis test was performed to test the relationship between the extract type factor and the germination percentage, GSI, RI, and hypocotyl and radicle length, followed by Dunn's multiple comparison test. The Kruskal–Wallis test and regression analysis were performed to investigate the effect of the extract concentrations. All statistical analyses were performed using SigmaPlot v12.0 (Systat Software Inc. Chicago, USA). The criterion for statistical significance was p < 0.05.

### In vivo bioassays in greenhouse

The soil used in the experiments was collected at Marafon Farm (latitude 9° 13′ 28,9″ South, longitude 44° 44′ 44,4″ West), Bom Jesus-PI, Brazil, in an area of native Cerrado, in the layer of 20 cm of depth and submitted to physical and chemical analysis (Table S9). The soil was air-dried, crushed, sieved in a 2 mm mesh sieve, packed in fiber bags, and autoclaved twice at 121 °C (1 atm) for 1 h with an interval of 24 h, which was later corrected according to recommendations for correctives and fertilization for the Cerrado^55^. For irrigation, the pots were weighed daily, and the volume of water in the soil was adjusted to 80% of the pot capacity.

Fifty *B. bipinnata* seeds were sown at a depth of 0.5 cm in plastic pots (19 cm in diameter) containing 3.5 kg of soil. Five days after the emergence of seedlings, the pots were thinned to five plants. At 20 days after sowing, treatments were performed using aqueous plant extracts at concentrations of 37.5, 75, 150, and 300 g L^−1^, and water was used as a control treatment. The experiment was carried out in CRD with 4 replications in a 4 × 4 + 1 factorial, the first factor being the extract type (DL, RC, PT, and JG) and the second the concentration.

The application of the extracts was carried out using a manual sprayer that delivered 200 L ha^−1^ at a spray pressure of 200 kPa. Flat nozzles were used in the sprayer. The phytotoxicity of the extracts on the weed was assessed daily for seven days based on the modified visual grading scale of the European Weed Research Council (EWRC)^37^, with grades from 1 to 7, where 1 represents no effect, 2 represents very slight, 3 represents slight, 4 represents moderate, 5 represents strong, 6 represents very strong, and 7 represents severe effects. The pots were kept in a greenhouse with an average temperature of 30 °C and a relative humidity of 60%. The variables seedling height and chlorophyll content were also measured.

Chlorophyll content was determined by ultraviolet–visible (UV–Vis) spectrophotometry of *B. bipinnata* leaves extracted with 80% acetone solution^56^. The absorbance of the samples was measured at wavelengths of 645, 646, and 663 nm with a single-beam UV–Vis spectrophotometer. Chlorophyll concentration was estimated following the Arnon equations^57^: Total chlorophyll (μg mL^−1^) = 20.2(A645) + 8.02(A663); Chlorophyll a (μg mL^−1^) = 12.7(A663) – 2.69(A645); Chlorophyll b (μg mL^−1^) = 22.9(A645) – 4.68(A663).

The Shapiro–Wilk test was performed to test the normality of the data. Normally distributed data were submitted to ANOVA followed by Tukey's test at 5% probability using the ExpDes package from R programming environment v.3.5.2 (R Core Team, Vienna, Austria). The data whose residues did not follow a normal distribution were submitted to the Kruskal–Wallis test followed by Dunn's multiple comparison test for the extract type factor and regression analysis for the doses using Sigma-Plot. The statistical significance criterion was p < 0.05.

### Determination of total phenolic content

The total phenolic contents of DL, RC, PT, and JG aqueous extracts were determined by UV–Vis spectroscopy using the Folin-Ciocalteu method with modifications^58^. A 100 µL aliquot of 2% (w/v) extract was mixed with 500 µL of Folin-Ciocateu reagent and 6 mL of distilled water and stirred for 1 min. Subsequently, 2 mL of 15% (w/v) Na_2_CO_3_ was added and stirred for 30 s, and then the volume was adjusted to 10 mL with distilled water. After 2 h at room temperature, the absorbance was measured at λ_max_ 750 nm using a UV–Vis spectrophotometer. The control sample was prepared with distilled water, following the same procedure described for the extracts. The calibration curve was constructed with gallic acid (10 to 350 µL mL^−1^) and expressed by the equation: C = 975.55A – 43.005; R = 0.992. The total phenol content was expressed as mg of GAE/g of dry weight. All analyses were performed in triplicate. Data were submitted to ANOVA and compared by Tukey's test at 5% probability using R software.

### ATR FT-MIR spectroscopy analysis combined with PCA

The infrared spectra of the aqueous extracts, previously dried in a lyophilizer, were acquired in an FTIR MIR/NIR spectrometer (PerkinElmer, Beaconsfield, BUCKS, UK) using a single reflectance horizontal MIRacle™ ATR cell (Pike Technologies, Madison, WI, USA) equipped with a zinc selenide crystal (ZnSe). Small amounts of the lyophilized extracts were deposited on the ATR crystal, and the spectra were obtained in the frequency range of 4000–550 cm^−1^, with a spectral resolution of 4 cm^−1^ and 32 scans. The ATR crystal was carefully cleaned with isopropyl alcohol, and the surface was allowed to dry before measuring the next sample. A background was recorded before each measurement and subtracted from the spectrum. Samples of plant species extracts were prepared in quadruplicate. The spectra were processed using Spectrum software (PerkinElmer, Shelton, CT, USA), in which automatic baseline correction, transformation of the spectral ordinate into absorbance and normalization were performed. Spectral data were subjected to PCA in the online software ChemoStat®^59^ using the spectral range of 1800–600 cm^−1^ and mean-centered data.

### Ethics and research guideline statement

Our study complies with relevant institutional, national, and international guidelines and legislation. Research permissions, including the collection of plant materials, were obtained by the Brazilian Biodiversity Information and Authorization System (SISBIO), Chico Mendes Institute for Biodiversity Conservation (ICMBio), Ministry of Environment (MMA) (SISBIO authorization number 83447-1).

## Supplementary Information

Supplementary Information.

## Data Availability

The datasets used and/or analyzed during the current study are available from the corresponding author on reasonable request.
