# Supplementary material for: Bioherbicidal potential of plant species with allelopathic effects on the weed Bidens bipinnata L
Source: Sci Rep. 2022 Aug 5;12:13476. doi: 10.1038/s41598-022-16203-5 (PMC9356026; doi:10.1038/s41598-022-16203-5)
Supplement: Supplementary file 1 — Supplementary Information. [file 41598_2022_16203_MOESM1_ESM.pdf]

Supplementary Information

Bioherbicidal potential of plant species with allelopathic effects on the weed *Bidens bipinnata* L.

Robson Willian Nunes Lopes, Estefenson Marques Morais, Julian Junio de Jesus Lacerda, Francisca Diana da Silva Araújo*

Campus Professora Cinobelina Elvas, Federal University of Piauí, 64900-000, Bom Jesus, PI, Brazil

*diana.araujo@ufpi.edu.br

Table S1. Seed germination percentage, germination speed index (GSI), allelopathic effect response index (RI), and hypocotyl and root length of *B. bipinnata* treated with different concentrations of *D. lacunifera* (DL), *R. communis* (RC), *P. tuberculatum* (PT), and *J. gossypiifolia* (JG) extracts.

Concentration g L^{-1}	DL	RC	PT	JG
	**Germination (%)**			
0	58.00	58.00	58.00	58.00
5	43.00	41.00	41.00	37.00
15	46.00	11.00	22.00	29.00
25	50.00	0.00	3.00	23.00
35	36.00	0.00	2.00	3.00
45	30.00	0.00	0.00	0.00
50	8.00	0.00	0.00	0.00
	**GSI**			
0	9.84	9.48	9.84	9.84
5	7.84	5.93	7.17	6.93
15	7.04	0.57	1.24	4.14
25	4.43	0.00	0.00	2.38
35	3.64	0.00	0.00	0.20
45	1.24	0.00	0.00	0.00
50	0.54	0.00	0.00	0.00
	**RI**			
0	0.00	0.00	0.00	0.00
5	-0.20	-0.30	-0.20	-0.40
15	-0.20	-0.90	-0.80	-0.60
25	-0.40	-1.00	-1.00	-0.70
35	-0.50	-1.00	-1.00	-0.70
45	-0.40	-1.00	-1.00	-1.00
50	-0.60	-1.00	-1.00	-1.00
	**Hypocotyl (mm)**			
0	17.61	17.61	17.61	17.61
5	12.08	9.40	19.41	19.70
15	13.50	0.00	0.00	8.32
25	6.60	0.00	0.00	0.00
35	5.46	0.00	0.00	0.00
45	0.00	0.00	0.00	0.00
50	0.00	0.00	0.00	0.00
	**Radicle (mm)**			
0	9.34	9.34	9.34	9.34
5	8.06	7.12	9.09	11.28
15	7.74	4.20	5.13	7.12
25	6.21	0.00	0.00	2.60
35	0.00	0.00	0.00	0.00
45	0.00	0.00	0.00	0.00
50	0.00	0.00	0.00	0.00

Table S2. Kruskal–Wallis test for the relationship between plant extract types used in the treatments of *B. bipinnata* and the variables germination percentage (G), germination speed index (GSI), allelopathic effect response index (RI), and hypocotyl and radicle length. DL: *D. lacunifera*; RC: *R. communis*; PT: *P. tuberculatum*; JG: *J. gossypifolia*.

Variable	Extracts	N	Missing	Median	25%	75%	df	H	P
G (%)	Control	4	0.00	54.00	52.00	64.00	4.00	33.52	<0.001
	DL	24	0.00	44.00	14.00	52.00			
	JG	24	0.00	8.00	0.00	30.00			
	PT	24	0.00	0.00	0.00	12.00			
	RC	24	0.00	0.00	0.00	10.00			
GSI	Control	4	0.00	9.39	8.46	11.24	4.00	29.94	<0.001
	DL	24	0.00	3.07	1.85	6.74			
	JG	24	0.00	0.54	0.00	4.06			
	PT	24	0.00	0.00	0.00	0.88			
	RC	24	0.00	0.00	0.00	0.47			
RI	Control	4	0.00	0.00	0.00	0.00	4.00	36.36	<0.001
	DL	24	0.00	-0.50	-0.80	0.00			
	JG	24	0.00	-0.50	-1.00	0.00			
	PT	24	0.00	-1.00	-1.00	-0.90			
	RC	24	0.00	-1.00	-1.00	-1.00			
Hypocotyl length (mm)	Control	4	0.00	18.28	15.76	19.47	4.00	18.75	<0.001
	DL	24	0.00	0.00	0.00	14.63			
	JG	24	0.00	0.00	0.00	0.00			
	PT	24	0.00	0.00	0.00	0.00			
	RC	24	0.00	0.00	0.00	0.00			
Radicle length (mm)	Control	4	0.00	9.59	8.36	10.34	4.00	9.90	0.042
	DL	24	0.00	0.00	0.00	9.32			
	JG	24	0.00	0.00	0.00	8.34			
	PT	24	0.00	0.00	0.00	3.07			
	RC	24	0.00	0.00	0.00	0.00			

df: degrees of freedom; H: Kruskal–Wallis statistics; P: probability.

Table S3. Dunn's test for multiple comparisons between treatments of *B. bipinnata* with *D. lacunifera* (DL), *R. communis* (RC), *P. tuberculatum* (PT), and *J. gossypifolia* (JG) extracts in *in vitro* bioassays using the variables germination percentage (G), germination speed index (GSI), allelopathic effect response index (RI), and hypocotyl and radicle length.

Variable	Comparison	Mean 1st Group	Mean 2nd Group	Median 1st Group	Median 2st Group	Diff of Ranks	Q	P<0.05
G (%)	Ctrl vs RC	58.00	9.04	54.00	0.00	55.04	3.513	Yes
	Ctrl vs PT	58.00	10.96	54.00	0.00	53.73	3.429	Yes
	Ctrl vs JG	58.00	16.00	54.00	8.00	44.29	2.827	Yes
	Ctrl vs DL	58.00	36.87	54.00	44.00	20.38	1.300	No
	DL vs RC	36.87	9.04	44.00	0.00	34.67	4.139	Yes
	DL vs PT	36.87	10.96	44.00	0.00	33.35	3.983	Yes
	DL vs JG	36.87	16.00	44.00	8.00	23.92	2.856	Yes
	JG vs RC	16.00	9.04	8.00	0.00	10.75	1.284	No
	JG vs PT	16.00	10.96	8.00	0.00	9.44	1.127	No
	PT vs RC	10.96	9.04	0.00	0.00	1.31	0.157	No
GSI	Ctrl vs RC	9.85	1.14	9.39	0.00	57.27	3.655	Yes
	Ctrl vs PT	9.85	1.53	9.39	0.00	54.17	3.457	Yes
	Ctrl vs JG	9.85	2.53	9.39	0.54	44.21	2.822	Yes
	Ctrl vs DL	9.85	4.41	9.39	3.07	24.56	1.568	No
	DL vs RC	4.41	1.14	3.07	0.00	32.71	3.906	Yes
	DL vs PT	4.41	1.53	3.07	0.00	29.60	3.535	Yes
	DL vs JG	4.41	2.53	3.07	0.54	19.65	2.346	No
	JG vs RC	2.53	2.53	0.54	0.00	13.06	1.56	No
	JG vs PT	2.53	1.53	0.54	0.00	9.96	1.189	No
	PT vs RC	1.53	1.14	0.00	0.00	3.10	0.371	No
RI	Ctrl vs RC	0.00	-0.93	0.00	-1.00	58.48	3.732	Yes
	Ctrl vs PT	0.00	-0.79	0.00	-1.00	48.31	3.084	Yes
	Ctrl vs JG	0.00	-0.48	0.00	-0.50	28.25	1.803	No
	Ctrl vs DL	0.00	-0.40	0.00	-0.50	21.21	1.354	No
	DL vs RC	-0.40	-0.93	-0.50	-1.00	37.27	4.45	Yes
	DL vs PT	-0.40	-0.79	-0.50	-1.00	27.10	3.236	Yes
	DL vs JG	-0.40	-0.48	-0.50	-0.50	7.04	0.841	No
	JG vs RC	-0.48	-0.93	-0.50	-1.00	30.23	3.609	Yes
	JG vs PT	-0.48	-0.79	-0.50	-1.00	20.06	2.396	No
	PT vs RC	-0.79	-0.93	-1.00	-1.00	10.17	1.214	No
Hypocotyl length (mm)	Ctrl vs RC	17.61	1.63	18.28	0.00	45.83	2.925	Yes
	Ctrl vs PT	17.61	3.33	18.28	0.00	42.56	2.717	No
	Ctrl vs JG	17.61	4.82	18.28	0.00	38.69	2.469	No
	Ctrl vs DL	17.61	6.55	18.28	0.00	31.25	1.995	No
	DL vs RC	6.55	1.63	0.00	0.00	14.58	1.741	No
	DL vs PT	6.55	3.33	0.00	0.00	11.31	1.351	No
	DL vs JG	6.55	4.82	0.00	0.00	7.44	0.888	No
	JG vs RC	4.82	1.63	0.00	0.00	7.15	0.853	No
	JG vs PT	4.82	3.33	0.00	0.00	3.88	0.463	No

	PT vs RC	3.33	1.63	0.00	0.00	3.27	0.391	No
Radicle length (mm)	Ctrl vs RC	9.35	1.97	9.59	0.00	38.08	2.431	No
	Ctrl vs PT	9.35	2.47	9.59	0.00	34.79	2.221	No
	Ctrl vs JG	9.35	3.65	9.59	0.00	30.81	1.967	No
	Ctrl vs DL	9.35	3.83	9.59	0.00	28.60	1.826	No
	DL vs RC	3.83	1.97	0.00	0.00	9.48	1.132	No
	DL vs PT	3.83	2.47	0.00	0.00	6.19	0.739	No
	DL vs JG	3.83	3.65	0.00	0.00	2.21	0.264	No
	JG vs RC	3.65	1.97	0.00	0.00	7.27	0.868	No
	JG vs PT	3.65	2.47	0.00	0.00	3.98	0.475	No
	PT vs RC	2.47	1.97	0.00	0.00	3.29	0.393	No

Ctrl: control treatment. Q: chi-squared.

Table S4. Kruskal–Wallis test for the relationship between extract concentrations used in the treatments of *B. bipinnata* and the variables germination percentage (G), germination speed index (GSI), allelopathic effect response index (RI), hypocotyl and radicle length. DL: *D. lacunifera*; RC: *R. communis*; PT: *P. tuberculatum*; JG: *J gossypifolia*.

Variable	Concentração (g L^{-1})	N	Missing	Median	25%	75%	df	H	P
G (%)	0	4.00	0.00	54.00	52.00	64.00	6.00	50.03	<0.001
	5	16.00	0.00	48.00	24.00	54.00			
	15	16.00	0.00	22.00	14.00	42.00			
	25	16.00	0.00	8.00	0.00	36.00			
	35	16.00	0.00	0.00	0.00	8.00			
	45	16.00	0.00	0.00	0.00	0.00			
	50	16.00	0.00	0.00	0.00	0.00			
GSI	0	4.00	0.00	9.39	8.46	11.24	6.00	60.15	<0.001
	5	16.00	0.00	7.35	4.93	9.48			
	15	16.00	0.00	1.76	0.57	6.21			
	25	16.00	0.00	1.25	0.00	2.83			
	35	16.00	0.00	0.00	0.00	1.18			
	45	16.00	0.00	0.00	0.00	0.00			
	50	16.00	0.00	0.00	0.00	0.00			
RI	0	4.00	0.00	0.00	0.00	0.00	6.00	32.75	<0.001
	5	16.00	0.00	-0.15	-0.45	0.00			
	15	16.00	0.00	-0.65	-0.90	-0.05			
	25	16.00	0.00	-0.90	-1.00	-0.60			
	35	16.00	0.00	-1.00	-1.00	-0.80			
	45	16.00	0.00	-1.00	-1.00	-0.90			
	50	16.00	0.00	-1.00	-1.00	-0.95			
Hypocotyl length (mm)	0	4.00	0.00	18.28	15.76	19.47	6.00	46.90	<0.001
	5	16.00	0.00	16.80	0.00	23.40			
	15	16.00	0.00	0.00	0.00	15.96			
	25	16.00	0.00	0.00	0.00	0.00			
	35	16.00	0.00	0.00	0.00	0.00			
	45	16.00	0.00	0.00	0.00	0.00			
	50	16.00	0.00	0.00	0.00	0.00			
Radicle length (mm)	0	4.00	0.00	9.59	8.36	10.34	6.00	52.60	<0.001
	5	16.00	0.00	11.80	0.00	13.57			
	15	16.00	0.00	7.43	0.00	9.32			
	25	16.00	0.00	0.00	0.00	3.40			
	35	16.00	0.00	0.00	0.00	0.00			
	45	16.00	0.00	0.00	0.00	0.00			
	50	16.00	0.00	0.00	0.00	0.00			

df: degrees of freedom; H: Kruskal–Wallis statistics; p: probability.

Table S5. Kruskal–Wallis test for the relationship between the concentrations of each extract used in the treatments of *B. bipinnata* seeds and the variables germination percentage (G), germination speed index (GSI), allelopathic effect response index (RI), hypocotyl and radicle length. DL: *D. lacunifera*; RC: *R. communis*; PT: *P. tuberculatum*; JG: *J gossypifolia*.

**Germination (%)**									
Extract	Concentration (g L^{-1})	N	Missing	Median	25%	75%	df	H	P
DL	0	4.00	0.00	54.00	52.00	64.00	6.00	13.75	0.033
	5	4.00	0.00	50.00	28.00	58.00			
	15	4.00	0.00	54.00	36.00	56.00			
	25	4.00	0.00	50.00	42.00	58.00			
	35	4.00	0.00	42.00	20.00	52.00			
	45	4.00	0.00	36.00	16.00	44.00			
	50	4.00	0.00	6.00	2.00	14.00			
JG	0	4.00	0.00	54.00	52.00	64.00	6.00	23.81	<0.001
	5	4.00	0.00	40.00	22.00	52.00			
	15	4.00	0.00	30.00	22.00	36.00			
	25	4.00	0.00	20.00	16.00	30.00			
	35	4.00	0.00	2.00	0.00	6.00			
	45	4.00	0.00	0.00	0.00	0.00			
	50	4.00	0.00	0.00	0.00	0.00			
PT	0	4.00	0.00	54.00	52.00	64.00	6.00	25.75	<0.001
	5	4.00	0.00	46.00	24.00	58.00			
	15	4.00	0.00	20.00	10.00	34.00			
	25	4.00	0.00	0.00	0.00	0.00			
	35	4.00	0.00	0.00	0.00	0.00			
	45	4.00	0.00	0.00	0.00	0.00			
	50	4.00	0.00	0.00	0.00	0.00			
RC	0	4.00	0.00	54.00	52.00	64.00	6.00	26.23	<0.001
	5	4.00	0.00	48.00	30.00	52.00			
	15	4.00	0.00	10.00	6.00	16.00			
	25	4.00	0.00	0.00	0.00	0.00			
	35	4.00	0.00	0.00	0.00	0.00			
	45	4.00	0.00	0.00	0.00	0.00			
	50	4.00	0.00	0.00	0.00	0.00			

**GSI**									
Extract	Concentration (g L^{-1})	N	Missing	Median	25%	75%	df	H	P
DL	0	4.00	0.00	9.39	8.46	11.24	6.00	18.74	0.005
	5	4.00	0.00	8.82	4.91	10.76			
	15	4.00	0.00	7.15	5.07	9.01			
	25	4.00	0.00	3.97	2.83	6.03			
	35	4.00	0.00	3.32	2.18	5.09			
	45	4.00	0.00	2.41	1.01	2.88			
	50	4.00	0.00	0.38	0.16	0.93			

JG	0	4.00	0.00	9.39	8.46	11.24	6.00	24.76	<0.001
	5	4.00	0.00	6.72	4.96	9.92			
	15	4.00	0.00	5.22	2.38	6.66			
	25	4.00	0.00	2.03	1.63	3.14			
	35	4.00	0.00	0.15	0.00	0.40			
	45	4.00	0.00	0.00	0.00	0.00			
	50	4.00	0.00	0.00	0.00	0.00			
PT	0	4.00	0.00	9.39	8.46	11.24	6.00	23.64	<0.001
	5	4.00	0.00	8.26	4.25	10.46			
	15	4.00	0.00	0.80	0.65	1.67			
	25	4.00	0.00	0.00	0.00	0.45			
	35	4.00	0.00	0.00	0.00	0.17			
	45	4.00	0.00	0.00	0.00	0.00			
	50	4.00	0.00	0.00	0.00	0.00			
RC	0	4.00	0.00	9.39	8.46	11.24	6.00	25.48	<0.001
	5	4.00	0.00	6.16	4.26	7.59			
	15	4.00	0.00	0.47	0.36	0.78			
	25	4.00	0.00	0.00	0.00	0.17			
	35	4.00	0.00	0.00	0.00	0.00			
	45	4.00	0.00	0.00	0.00	0.00			
	50	4.00	0.00	0.00	0.00	0.00			

RI

Extract	Concentration ($g\ L^{-1}$)	N	Missing	Median	25%	75%	df	H	P
DL	0	4.00	0.00	0.00	0.00	0.00	6.00	10.87	0.092
	5	4.00	0.00	0.00	-0.05	0.00			
	15	4.00	0.00	-0.20	-0.55	0.00			
	25	4.00	0.00	-0.40	-0.75	-0.05			
	35	4.00	0.00	-0.65	-0.80	-0.30			
	45	4.00	0.00	-0.70	-0.90	-0.30			
	50	4.00	0.00	-0.95	-1.00	-0.45			
JG	0	4.00	0.00	0.00	0.00	0.00	6.00	9.08	0.169
	5	4.00	0.00	-0.10	-0.35	0.00			
	15	4.00	0.00	-0.30	-0.55	-0.05			
	25	4.00	0.00	-0.65	-0.85	-0.25			
	35	4.00	0.00	-0.95	-1.00	-0.45			
	45	4.00	0.00	-1.00	-1.00	-0.50			
	50	4.00	0.00	-0.50	-1.00	0.00			
PT	0	4.00	0.00	0.00	0.00	0.00	6.00	24.14	<0.001
	5	4.00	0.00	-0.15	-0.60	0.25			
	15	4.00	0.00	-0.80	-0.90	-0.35			
	25	4.00	0.00	-1.00	-1.00	-0.95			
	35	4.00	0.00	-1.00	-1.00	-1.00			
	45	4.00	0.00	-1.00	-1.00	-1.00			
	50	4.00	0.00	-1.00	-1.00	-1.00			
RC	0	4.00	0.00	0.00	0.00	0.00	6.00	22.29	0.001

	5	4.00	0.00	-0.60	-0.90	-0.30			
	15	4.00	0.00	-1.00	-1.00	-0.95			
	25	4.00	0.00	-1.00	-1.00	-1.00			
	35	4.00	0.00	-1.00	-1.00	-1.00			
	45	4.00	0.00	-1.00	-1.00	-1.00			
	50	4.00	0.00	-1.00	-1.00	-1.00			

Hypocotyl length (mm)

Extract	Concentration (g L^{-1})	N	Missing	Median	25%	75%	df	H	P
DL	0	4.00	0.00	18.28	15.76	19.47	6.00	16.72	0.010
	5	4.00	0.00	15.55	7.36	16.80			
	15	4.00	0.00	17.12	8.30	18.71			
	25	4.00	0.00	5.94	0.00	13.21			
	35	4.00	0.00	4.98	0.00	10.92			
	45	4.00	0.00	0.00	0.00	0.00			
	50	4.00	0.00	0.00	0.00	0.00			
JG	0	4.00	0.00	18.28	15.76	19.47	6.00	19.15	0.004
	5	4.00	0.00	22.67	11.32	27.43			
	15	4.00	0.00	7.66	0.00	16.63			
	25	4.00	0.00	0.00	0.00	0.00			
	35	4.00	0.00	0.00	0.00	0.00			
	45	4.00	0.00	0.00	0.00	0.00			
	50	4.00	0.00	0.00	0.00	0.00			
PT	0	4.00	0.00	18.28	15.76	19.47	6.00	21.92	0.001
	5	4.00	0.00	24.73	12.05	26.23			
	15	4.00	0.00	0.00	0.00	0.00			
	25	4.00	0.00	0.00	0.00	0.00			
	35	4.00	0.00	0.00	0.00	0.00			
	45	4.00	0.00	0.00	0.00	0.00			
	50	4.00	0.00	0.00	0.00	0.00			
RC	0	4.00	0.00	18.28	15.76	19.47	6.00	20.49	0.002
	5	4.00	0.00	7.50	0.00	18.80			
	15	4.00	0.00	0.00	0.00	0.00			
	25	4.00	0.00	0.00	0.00	0.00			
	35	4.00	0.00	0.00	0.00	0.00			
	45	4.00	0.00	0.00	0.00	0.00			
	50	4.00	0.00	0.00	0.00	0.00			

Radicle length (mm)

Extract	Concentration (g L^{-1})	N	Missing	Median	25%	75%	df	H	P
DL	0	4.00	0.00	9.59	8.36	10.34	6.00	16.61	0.011
	5	4.00	0.00	9.98	4.78	11.36			
	15	4.00	0.00	9.92	4.54	10.96			
	25	4.00	0.00	7.57	3.40	9.03			
	35	4.00	0.00	0.00	0.00	0.00			
	45	4.00	0.00	0.00	0.00	0.00			

	50	4.00	0.00	0.00	0.00	0.00			
JG	0	4.00	0.00	9.59	8.36	10.34	6.00	16.62	0.011
	5	4.00	0.00	14.55	6.87	15.71			
	15	4.00	0.00	8.34	3.78	10.48			
	25	4.00	0.00	0.00	0.00	5.21			
	35	4.00	0.00	0.00	0.00	0.00			
	45	4.00	0.00	0.00	0.00	0.00			
	50	4.00	0.00	0.00	0.00	0.00			
PT	0	4.00	0.00	9.59	8.36	10.34	6.00	20.30	0.002
	5	4.00	0.00	11.86	5.64	12.54			
	15	4.00	0.00	6.42	3.07	7.20			
	25	4.00	0.00	0.00	0.00	0.00			
	35	4.00	0.00	0.00	0.00	0.00			
	45	4.00	0.00	0.00	0.00	0.00			
	50	4.00	0.00	0.00	0.00	0.00			
RC	0	4.00	0.00	9.59	8.36	10.34	6.00	16.59	0.011
	5	4.00	0.00	6.70	0.00	14.25			
	15	4.00	0.00	3.65	0.00	8.40			
	25	4.00	0.00	0.00	0.00	0.00			
	35	4.00	0.00	0.00	0.00	0.00			
	45	4.00	0.00	0.00	0.00	0.00			
	50	4.00	0.00	0.00	0.00	0.00			

df: degrees of freedom; H: Kruskal–Wallis statistics; P: probability.

Table S6. Phytotoxicity indices in *B. bipinnata* leaves treated with increasing doses of *D. lacunifera* (DL), *R. communis* (RC), *P. tuberculatum* (PT), and *J. gossypiifolia* (JG) extracts.

**Concentration ($g\ L^{-1}$)**	**Phytotoxicity**			
	**DL**	**RC**	**PT**	**JG**
0	1 no effect	1 no effect	1 no effect	1 no effect
37.5	3 slight	5 strong	5 strong	1 no effect
75	2 very slight	4 moderate	4 moderate	1 no effect
150	3 slight	4 moderate	4 moderate	1 no effect
300	2 very slight	4 moderate	4 moderate	1 no effect

Table S7. Height, chlorophyll a, b, and total of *B. bipinnata* plants treated with different concentrations of *D. lacunifera* (DL), *R. communis* (RC), *P. tuberculatum* (PT), and *J. gossypiifolia* (JG) extracts.

Concentration g L^{-1}	DL	RC	PT	JG
	**Height (cm)**			
0	21.43	21.43	21.43	21.43
37.5	13.43	8.45	7.75	13.00
75	11.68	9.15	8.55	13.75
150	10.20	10.88	7.58	15.00
300	10.30	9.45	8.13	11.50
	**Chlorophyll a (µg mL^{-1})**			
0	21.51	21.51	21.51	21.51
37.5	18.45	18.78	7.54	18.33
75	18.22	8.15	12.64	16.09
150	16.45	12.27	7.92	13.07
300	10.88	10.30	8.62	8.21
	**Chlorophyll b (µg mL^{-1})**			
0	12.07	12.07	12.07	12.07
37.5	10.03	3.03	5.59	6.21
75	11.29	0.94	3.28	9.86
150	6.49	4.87	5.91	5.07
300	9.47	1.37	6.77	6.70
	**Total chlorophyll (µg mL^{-1})**			
0	26.94	26.94	26.94	26.94
37.5	16.38	12.91	13.13	21.96
75	19.65	9.09	13.26	19.79
150	15.77	17.14	13.83	18.14
300	17.52	11.67	15.39	14.91

Table S8. Analysis of variance for height and chlorophyll a of *B. bipinnata* plants treated with different concentrations of *D. lacunifera* (DL), *R. communis* (RC), *P. tuberculatum* (PT), and *J. gossypiifolia* (JG) extracts by *in vivo* bioassays.

Variable	Extracts	N	Mean	df	F	P
Plant height	DL	16	11.40ab	3	8.135	<0.001
	RC	16	9.48bc			
	PT	16	8.00c			
	JG	16	13.31a			
	Conc					
	37.5	4	10.66	3	0.353	0.7869
	75	4	10.78			
	150	4	10.91			
	300	4	10.65			
	Extracts * Conc	68		9	0.5994	0.7915
	Ctrl vs Factorial					
	Ctrl	4	21.43a	1	42.459	<0.001
	Factorial	64	10.54b			
Chlorophyll a	DL	16	16.00a	3	3.683	0.0177
	JG	16	13.93ab			
	RC	16	9.87ab			
	PT	16	9.18b			
	Conc					
	37.5	4	13.27	3	1.263	0.297
	75	4	13.77			
	150	4	12.43			
	300	4	9.50			
	Extracts * Conc	68		9	0.747	0.6645
	Ctrl vs Factorial					
	Ctrl	4	21.51a	1	6.981	0.0109
	Factorial	64	12.25 b			

Ctrl: control treatment. Conc.: concentration.

Table S9. Kruskal–Wallis test and Dunn's multiple comparison test for chlorophyll b and total of *B. bipinnata* plants treated with different concentrations of *D. lacunifera* (DL), *R. communis* (RC), *P. tuberculatum* (PT), and *J. gossypiifolia* (JG) extracts in *in vivo* bioassays.

**Kruskal–Wallis test**										
Variable	Extracts	N	Missing	Mean	Median	25%	75%	df	H	P
Chlorophyll b	Ctrl	4	0.00	12.07	9.97	2.89	21.25	4.00	14.3	0.006
	DL	16	0.00	9.32	7.26	2.33	12.80			
	RC	16	0.00	2.55	1.44	0.69	3.41			
	PT	16	0.00	5.39	1.58	0.80	10.26			
	JG	16	0.00	6.96	5.27	3.25	8.31			
Total chlorophyll	Ctrl	4	0.00	12.07	24.82	20.56	33.31	4.00	11.892	0.018
	DL	16	0.00	9.32	14.63	12.27	20.29			
	RC	16	0.00	2.55	9.82	7.67	16.77			
	PT	16	0.00	5.39	12.07	9.83	18.02			
	JG	16	0.00	6.96	19.59	12.89	23.86			

**Dunn's test**								
Variable	Comparison	Mean 1st Group	Mean 2nd Group	Median 1st Group	Median 2st Group	Diff of Ranks	Q	$p<0.05$
Chlorophyll b	Ctrl vs RC	12.07	2.55	9.97	1.44	25.47	2.30	No
	Ctrl vs PT	12.07	5.39	9.97	1.58	16.34	1.48	No
	Ctrl vs JG	12.07	6.96	9.97	5.27	5.91	0.53	No
	Ctrl vs DL	12.07	9.32	9.97	7.26	3.28	0.30	No
	DL vs RC	9.32	2.55	7.26	1.44	22.19	3.17	No
	DL vs PT	9.32	5.39	7.26	1.58	13.06	1.87	No
	DL vs JG	9.32	6.96	7.26	5.27	2.63	0.38	No
	JG vs RC	6.96	2.55	5.27	1.44	19.56	2.80	No
	JG vs PT	6.96	5.39	5.27	1.58	10.44	1.49	No
	PT vs RC	5.39	2.55	1.58	1.44	9.13	1.31	No
Total chlorophyll	Ctrl vs RC	26.94	12.70	24.82	9.82	31.38	2.84	Yes
	Ctrl vs PT	26.94	13.90	24.82	12.07	25.75	2.33	No
	Ctrl vs DL	26.94	17.33	24.82	14.63	19.00	1.72	No
	Ctrl vs JG	26.94	18.70	24.82	19.59	14.19	1.28	No
	JG vs RC	18.70	12.70	19.59	9.82	17.19	2.46	No
	JG vs PT	18.70	13.90	19.59	12.07	11.56	1.65	No
	JG vs DL	18.70	17.33	19.59	14.63	4.81	0.69	No
	DL vs RC	17.33	12.70	14.63	9.82	12.38	1.77	No
	DL vs PT	17.33	13.90	14.63	12.07	6.75	0.97	No
	PT vs RC	13.90	12.70	12.07	9.82	5.63	0.81	No

df: degrees of freedom; H: Kruskal–Wallis statistics; P: probability. Ctrl: control treatment. Q: chi-squared.

Table S10. Kruskal–Wallis test for the relationship between concentrations of each extract used in the treatments of *B. bipinnata* seeds and the variables germination percentage (G), germination speed index (GSI), allelopathic effect response index (RI), hypocotyl and radicle length in *in vivo* bioassays. DL: *D. lacunifera*; RC: *R. communis*; PT: *P. tuberculatum*; JG: *J gossypifolia*.

**Chlorophyll b**									
Variable	Conc. (g L^{-1})	N	Missing	Median	25%	75%	df	H	P
DL	0	4	0.00	9.97	2.89	21.25	4.00	1.471	0.832
	37.5	4	0.00	10.24	7.26	12.80			
	75	4	0.00	7.86	4.12	18.46			
	150	4	0.00	5.68	1.41	11.58			
	300	4	0.00	3.21	2.00	16.94			
RC	0	4	0.00	9.97	2.89	21.25	4.00	8.843	0.065
	37.5	4	0.00	3.34	1.92	4.15			
	75	4	0.00	0.92	0.44	1.44			
	150	4	0.00	2.40	1.51	8.24			
	300	4	0.00	0.60	0.17	2.58			
PT	0	4	0.00	9.97	2.89	21.25	4.00	3.7	0.448
	37.5	4	0.00	5.72	1.01	10.17			
	75	4	0.00	1.24	0.71	5.87			
	150	4	0.00	5.24	0.80	11.02			
	300	4	0.00	5.94	0.91	12.63			
JG	0	4	0.00	9.97	2.89	21.25	4.00	0.614	0.961
	37.5	4	0.00	5.06	4.14	8.28			
	75	4	0.00	5.26	3.30	16.43			
	150	4	0.00	4.83	1.84	8.31			
	300	4	0.00	5.27	3.84	9.56			
**Total chlorophyll**									
Variable	Conc. (g L^{-1})	N	Missing	Median	25%	75%	df	H	P
DL	0	4	0.00	24.82	20.56	33.31	4.00	4.7	0.319
	37.5	4	0.00	17.21	12.56	20.19			
	75	4	0.00	19.13	13.91	25.38			
	150	4	0.00	15.68	12.33	19.21			
	300	4	0.00	10.68	4.76	30.29			
RC	0	4	0.00	24.82	20.56	33.31	4.00	7.116	0.13
	37.5	4	0.00	8.89	7.83	18.00			
	75	4	0.00	9.56	6.74	11.44			
	150	4	0.00	16.77	11.06	23.22			
	300	4	0.00	7.47	6.32	17.02			
PT	0	4	0.00	24.82	20.56	33.31	4.00	6.271	0.18
	37.5	4	0.00	14.68	8.37	17.88			
	75	4	0.00	11.32	9.97	16.56			
	150	4	0.00	12.93	9.45	18.21			
	300	4	0.00	14.24	10.06	20.72			

	0	4	0.00	24.82	20.56	33.31	4.00	4.014	0.404
	37.5	4	0.00	19.98	13.71	30.21			
JG	75	4	0.00	21.12	14.15	25.42			
	150	4	0.00	17.83	13.64	22.65			
	300	4	0.00	14.23	8.47	21.36			

df: degrees of freedom; H: Kruskal–Wallis statistics; P: probability. Conc.: concentration.

Table S11. Assignment of ATR FT-MIR spectra of leaf extracts of *D. lacunifera*, *R. communis*, *P. tuberculatum*, and *J. gossypiifolia*.

**Wavenumber (cm^{-1})**	**Base group and vibration mode**	**Main attribution**
3284, 3174	υ (O−H)	Alcohol, carboxilic acid, phenol
2934, 2931, 2926, 2920	$υ_{as}$ (C−H)	Alkane
1732	υ (C=O)	Ester, ketone, aldehyde
1595, 1594, 1590	υ (N−H), υ (−C=C−)	Amine, alkene, aromatic
1404, 1387, 1368	δ (C−H)	Alkane
1254, 1253	$υ_{as}$(C—O)	Alcohol
1189	υ (C—O), δ (C—OH)	Carboxilic acid, alcohol
1049, 1043	υ (C−O−C), υ (C−C), δ (C−OH)	Aromatic, alcohol
950 – 675	γ (C−H)	Alkene, aromatic

υ, stretching or vibration; δ, in-plane deformation; γ, out-of-plane deformation; s, symmetrical; as, asymmetrical.

Table S12. Physicochemical characterization of the soil used in the bioassay in the greenhouse.

pH	H+Al	Al	Ca	Mg	K	SB	T	P	
(H_2O)	-------------------------- cmolc dm^{-3} --------------------------							mg dm^{-3}	
4.7	8.3	1.8	0.02	0.01	0.01	0.04	8.38	1.87	
**Fe**	**Cu**	**Mn**	**Zn**	**V**	**m**	**MO**	**Clay**	**Silt**	**Sand**
------------ mg dm^{-3} ----------				--- % ----		g kg^{-1}	-------- g kg^{-1} -------		
135	0.19	1.85	0.01	0.5	97.9	24.1	244	11	753

H+Al: potential acidity; SB: sum of exchangeable bases; T: cation exchange capacity at pH 7.0; V: base saturation index; m: aluminum saturation index; and MO: organic matter.

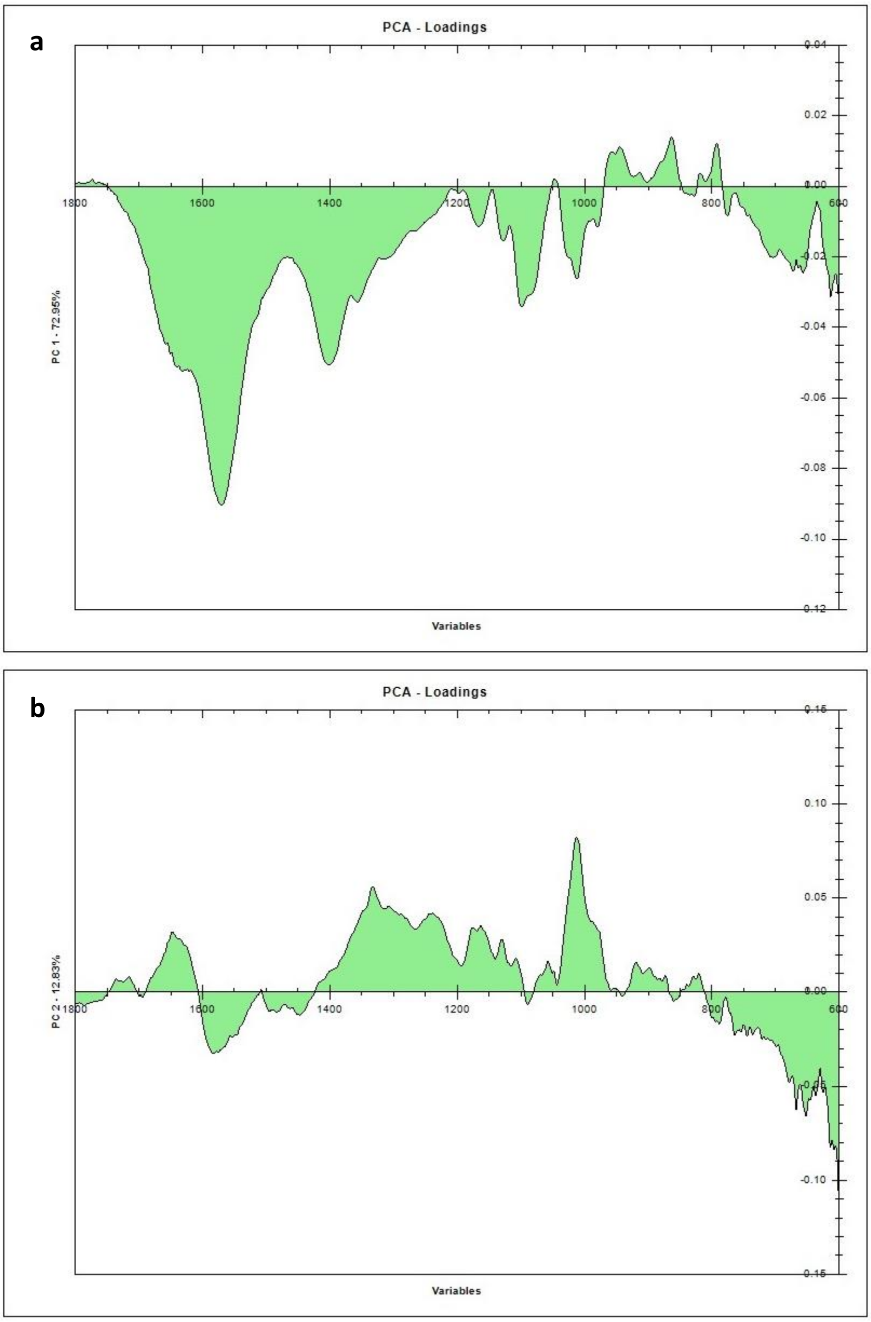

Figure S1. PC1 (a) and PC2 (b) loading plots of *D. lacunifera*, *R. communis*, *P. tuberculatum*, and *J. gossypiifolia* extracts.
